# Limits and Prospects of Molecular Fingerprinting for Phenotyping Biological Systems Revealed through *In Silico* Modeling

**DOI:** 10.1021/acs.analchem.2c04711

**Published:** 2023-04-12

**Authors:** Tarek Eissa, Kosmas V. Kepesidis, Mihaela Zigman, Marinus Huber

**Affiliations:** † Department of Laser Physics, Ludwig Maximilian University of Munich (LMU), 85748 Garching, Germany; ‡ Laboratory for Attosecond Physics, Max Planck Institute of Quantum Optics (MPQ), 85748 Garching, Germany; § Department of Informatics, Technical University of Munich (TUM), 85748 Garching, Germany

## Abstract

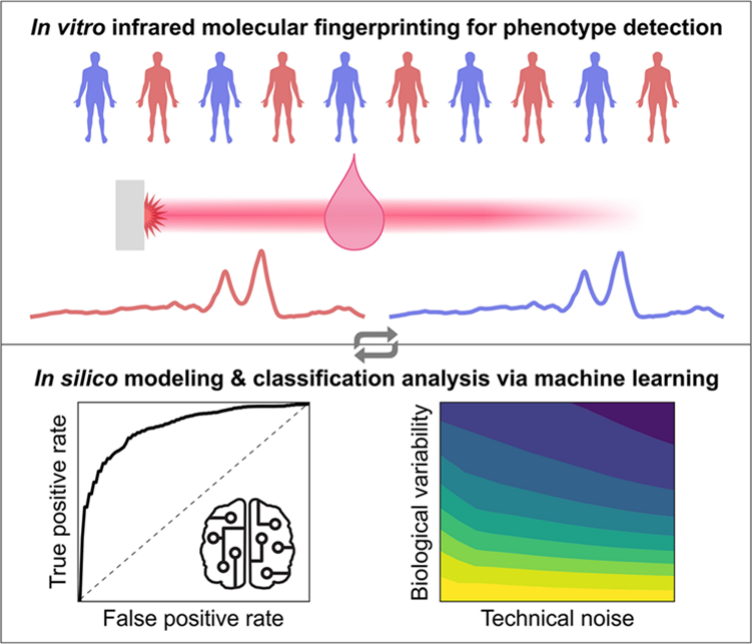

Molecular fingerprinting via vibrational spectroscopy characterizes the chemical composition of molecularly complex media which enables the classification of phenotypes associated with biological systems. However, the interplay between factors such as biological variability, measurement noise, chemical complexity, and cohort size makes it challenging to investigate their impact on how the classification performs. Considering these factors, we developed an *in silico* model which generates realistic, but configurable, molecular fingerprints. Using experimental blood-based infrared spectra from two cancer-detection applications, we validated the model and subsequently adjusted model parameters to simulate diverse experimental settings, thereby yielding insights into the framework of molecular fingerprinting. Intriguingly, the model revealed substantial improvements in classifying clinically relevant phenotypes when the biological variability was reduced from a between-person to a within-person level and when the chemical complexity of the spectra was reduced. These findings quantitively demonstrate the potential benefits of personalized molecular fingerprinting and biochemical fractionation for applications in health diagnostics.

Vibrational spectroscopy by means of Raman or infrared techniques is a powerful analytical platform capable of characterizing molecular samples at any state of matter in a label-free manner.^1,2^ Since essentially every molecule exhibits a unique vibrational spectrum, spectroscopic approaches are able to quantify individual molecular contributions in complex matrices.^3^ Although determining changes in the nature and quantity of individual molecular species is challenged by overlapping spectral bands, the vibrational spectrum still reflects the overall molecular composition of a given sample and is therefore referred to as its “molecular fingerprint”. Statistical or machine learning methods can identify spectral patterns specific to molecular phenotypes and consequently classify samples.

Molecular fingerprinting by vibrational spectroscopy has been increasingly applied to biomedical problems.^4^ The approach has been used to classify bacteria and cell (sub)types,^5,6^ distinguish between benign and malignant tissues,^1,2^ and identify diseases based on fingerprint spectra of biofluids.^2,7^

Although successfully applied, the prospects and fundamental limitations of vibrational fingerprinting for certain applications, such as for clinically relevant questions, remain largely unexplored.^2,8^ The challenge is that a variety of technical and data acquisition aspects impact the measured spectra and thus can affect the classification accuracy.^9−11^ At the same time, every living system exhibits an inherent level of biological variability,^10,12,13^ further challenging the unambiguous identification of different molecular states. While previous work has examined the variations caused by technical, data acquisition, and biological aspects on recorded spectra,^10,11,14^ disentangling their individual contribution on the classification accuracy remains experimentally challenging. This often requires large-scale studies involving different measurement instruments, different laboratories, and different protocols for sample handling, which may be difficult to realize due to resource limitations.^15^

*In silico* investigations performed via computer simulations are particularly powerful in this respect as they can address problems that are practically or experimentally challenging.^16,17^ By creating computational models capable of mimicking the behavior of biological systems, considering various sources of noise or heterogeneity, one can rapidly gain insights into the underlying mechanisms that define the behavior of a system under differing simulated conditions.^18^

To investigate the fundamental capacity of vibrational fingerprinting, we propose an *in silico* approach that generates artificial infrared spectra (Figure 1). Our approach is based on modeling the molecular composition of a given biological system in a defined molecular state and transforming this composition into a measurable quantity—the resulting infrared spectrum. With this model, analytical sources of variation can be considered to obtain realistic spectra that have comparable properties to experimental observations. Not only does this allow for the generation of simulated “measurement events” in any number, but also enables the precise control of crucial parameters that may impact the recorded spectra. Our model provides foundational insights into the factors affecting the underlying measurement approach—molecular fingerprinting of complex biological systems—and carries the capacity to guide future experiments without the need for exhaustive sample collections and measurements.

**Figure 1:**
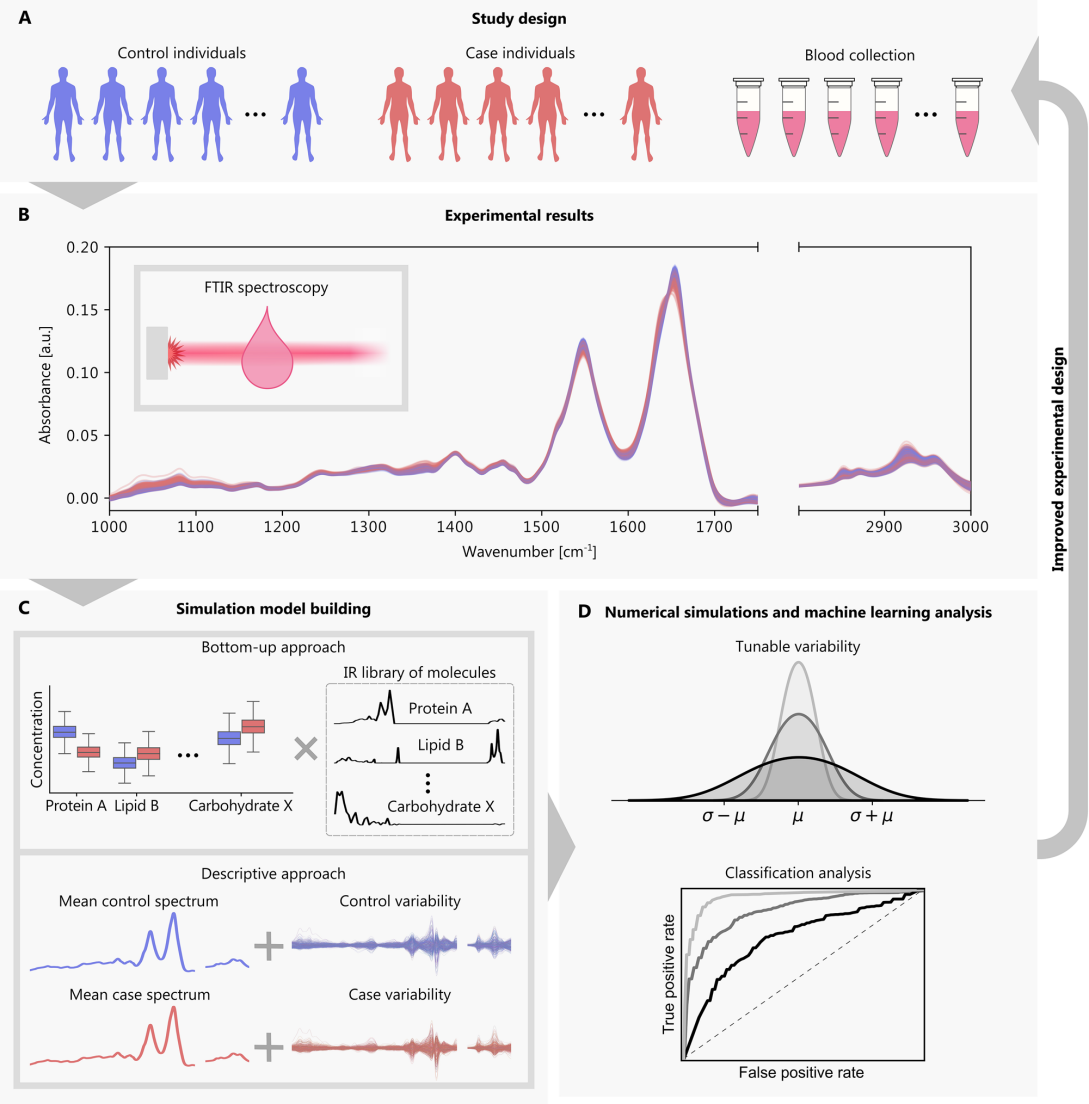
Overview of the *in silico* model and its application to infrared fingerprinting for disease detection. (A) Blood samples are collected from case and control individuals, and (B) blood-based samples are measured with an infrared spectrometer. Machine learning algorithms are applied to the spectral data set, and a value of classification accuracy is retrieved. (C) Same problem can be investigated with an *in silico* model using either a bottom-up approach based on single component spectra or a descriptive approach based on measured infrared fingerprints of blood-based samples. (D) Within the frame of the model, the influence of simulated experimental conditions is investigated to gain insight into the effects different simulated conditions have on the predictive capacity of machine learning models. Based on these findings, (A) initial study design or (B) measurement mode can be adapted to improve the performance of the envisioned application.

The model is described and tested on a previously published application—infrared fingerprinting of blood sera to detect lung and prostate cancer.^7^ Our descriptions are kept as general as possible to facilitate their transferability to other fingerprinting techniques and applications. Furthermore, we provide a toolbox (written in Python),^19^ including the generated data, to allow for convenient applications of the proposed model. Although applied here to infrared spectra, the model can be applied to other molecular fingerprinting approaches, such as Raman^20^ or nuclear magnetic resonance^21^ spectroscopy.

Altogether, we obtain excellent agreement between the results of experimental and simulated data. By systematically adjusting all model parameters, we explored the potentials and limitations of molecular fingerprinting and thus contribute to accelerating its real-world applications.

## Methods

### *In Silico* Model

In essence, the problems tackled by molecular pattern recognition using machine learning are often very related. Depending on the application, by detecting changes in qualitative or quantitative aspects, a distinction between two or more molecular states is desired. In practice, this distinction is only possible to a certain extent due to the inherent biological or sample variability as well as measurement-specific errors. Additionally, often a limited amount of data is available to robustly validate conclusions drawn by analyzing differences between the studied states. Therefore, it can be difficult to make conclusive statements about the potential and limitations of the method in question or to isolate the molecular signal of interest. Even in a scenario where the signal is already known (e.g., the spectrum of a specific molecule), the expected accuracy of distinguishing between different states often requires numerous measurements due to the multiple sources of variation. To facilitate the investigation of these points, the *in silico* model should be able to generate spectra which meet the following requirements:

- Reflect the biological or sample variability of a given sample pool.
- Incorporate differences between different molecular states (e.g., cases and controls).
- Consider characteristics of the physical measurement, namely the noise introduced by the measurement device.

We describe two approaches to generate the artificial spectra. In a so-called bottom-up approach, the contribution of individual molecular species in a given sample is considered. Although intuitive, this approach cannot be implemented in the case of infrared spectroscopy of highly complex samples as we discuss below. Instead, it provides the motivation and basis for implementing another so-called descriptive approach which follows a similar mathematical formulation. Although the model can be applied to study different samples types with varying chemical complexities and to several molecular fingerprinting techniques, infrared molecular fingerprints of blood serum will serve as the guiding principle for assembling the model.

### Bottom-Up Approach

It has been shown that by breaking down a molecularly complex sample into its individual components, the corresponding infrared spectrum can be reasonably well approximated with a linear combination of individual component spectra of the most abundant molecular species.^22^ Thereby, each molecular spectrum ***x***_*i*_ is scaled to its respective concentration *c_i_* with ∑_*i* = 1_^*M*^*c*_*i*_ · ***x***_*i*_.

This idea can be also utilized to create spectra of a given population. The normal concentration ranges for the most abundant blood components in healthy individuals—which in total make up more than 99% of the molecular mass—can be obtained from the literature.^23,24^ Using these facts, the concentration parameter *c_i_* could be replaced with a random variable *c*(μ_*i*_, σ_*i*_) that models the distribution of each component for a healthy cohort. Thereby, a set of spectra that model the molecularly complex samples could be simulated where each spectrum is modeled as a statistical outcome ***Y*** with:
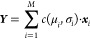
1

We term this model formulation as the bottom-up approach.

It is important to note that the bottom-up approach contains several simplifications and assumptions. Specifically, it is assumed that the concentrations of individual molecules or molecular classes are independent, which is not the case. In reality, biological networks are interconnected, and molecular changes within living organisms are occurring in a correlated fashion.^25^ In cases where these correlations are well known, introducing dependencies between the corresponding random variables would, in principle, account for this behavior.

The assumption that an infrared spectrum of a complex sample can be described by a linear combination of individual component spectra scaled to their concentrations can be regarded as sufficiently fulfilled. Although molecular spectra are influenced by inter-molecular interactions^26,27^ and environmental conditions (e.g., temperature and pH-value), several studies suggest that complex blood spectra can be described by linear superposition of individual single component spectra. For infrared spectroscopy, it is known that the strength of a molecular spectrum scales linearly with concentration over many orders of magnitude.^3,28^ Furthermore, it has been shown that the concentration of several different molecules in blood can be determined by linear regression.^29−32^ Moreover, it was shown that a linear combination of the spectra of the most abundant molecular species can be used to describe experimentally observed differences in the spectra of blood serum from a healthy cohort and from patients with lung cancer.^22^

While the bottom-up approach is straightforward to envision, access to the associated molecular spectra is essential for creating the model. This is a crucial limitation since, to our knowledge, there exists no consistent database with infrared spectra of the majority of molecules contained in complex biological systems. While previous work suggests that 12 selected protein spectra can model the shape of blood serum spectra,^22^ our results show that significantly more are required to represent the molecular complexity and biological variability adequately. Taken together, from the current perspective, it is unlikely that the bottom-up can be implemented when studying a complex blood-based matrix. In principle, however, it can be used to simulate the spectra of simpler systems, such as for pharmaceutical samples that can be described as a mixture of a few well-known substances.^33^

### Descriptive Approach

To mitigate the limited access to individual component infrared spectra, we introduce an alternative descriptive approach. Here, we use a set of *m* experimentally derived spectra ***s***_*i*_ that describe the samples of a particular biological matrix (e.g., blood serum of healthy individuals). By utilizing a random variable β(μ_*i*_, σ_*i*_) assuming a Gaussian distribution, the spectrum of a measured biological matrix can now be expressed as another statistical outcome ***Y*** with:
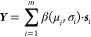
2

Thereby, the mathematical structure of the formulation is similar to the bottom-up approach, but utilizing a different set of calibration vectors to realize the model.

One difference between the two model formulations is that we cannot rely on the literature to obtain the concentration ranges for individual molecules and construct a realistic behavior for the biological variability. Therefore, a calibration procedure is required to adjust the statistical variables and select the experimentally derived spectra such that the resulting spectra reflect the biological variability of the measured samples. In addition, we must validate that assuming a Gaussian distribution creates spectral cohorts that adequately match experimentally measured cohorts.

To calibrate the biological variability, we use a set of experimentally measured blood serum spectra ***b***_*i*_ and calculate the expected value and variance of the descriptive approach:
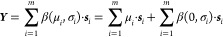
3
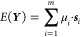
4
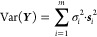
5Under the condition that the expected value *E*(***Y***) should correspond to the mean of the experimentally measured spectra ***b*®**, we can replace the sum of the scaled vectors ∑_*i*=1_^*m*^ μ_*i*_ · ***s***_*i*_ in eqs 3 and 4 by ***b*®**. Additionally, by comparing the variance of eq 5 to the sample variance , we set  and ***s***_*i*_ = ***b***_*i*_ – ***b*®**. With this, the expected value and variance of the generated spectra become equal to the experimentally determined values.

Since the considered number of measured spectra *m* may be smaller than the number of different molecular species *M* within the sample, this alternative representation may not be able to reproduce the complete molecular complexity. However, considering that we can often use hundreds of measurements for modeling, this approach should be able to capture variations related to a similar number of molecular species and thus asymptotically approximate most of the biological variability.

### Incorporating Measurement Errors

In addition to modeling the biological properties, it is important to factor in the contributions of noise introduced by the measurement device to assess its influence on the application of interest.

When using a commercial Fourier-transform infrared (FTIR) spectrometer, the dominant noise type inherent to the spectral measurement itself is additive white noise. Such noise can be added to a modeled spectrum as a vector **ϵ**:
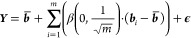
6

To determine a realistic estimate for **ϵ**, we repeatedly measured water samples with the FTIR device used for the blood sera measurements and calculated the standard deviation observed across the spectral features (Figure S1 in the Supporting Information). The measurement noise coefficient **ϵ** was thus calibrated to be a random Gaussian vector with a mean 0 and a spectrally dependent standard deviation.

Other sources of error in infrared spectroscopy which result from baseline drift^3^ or sample delivery and preparation^10,14^ can also be considered by introducing multiplicative noise and drift vectors in the model.^28^ When transferring this model to Raman-based fingerprinting applications, it may be necessary to consider further, partly nonlinear noise sources^34^ to obtain realistic results. As we show below, for the application of the model presented in this work, it is sufficient to consider only additive white noise to obtain realistic results.

### Incorporating Differences between Molecular States

Changes in the physiology of an organism, e.g., due to the onset of a disease, may change the molecular composition of the analyzed sample. Thus, we can choose a new set of experimentally measured spectra ***b***_i_^*^ that characterize the new molecular state Y* and use that as a basis for calibration. As later shown, for certain applications, the discriminant features can be largely explained by a single vector ***d***. By utilizing an additional Gaussian variable δ(μ_d_, σ_d_) which scales the introduced vector ***d*** and accounts for its variability, a modeled spectrum of an alternative class can be reduced to:
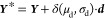
7

With this modeling platform, an arbitrary number of samples can be generated for different molecular states, resulting in a generated cohort that reflects the statistical properties of the measured sample pool ***b***_1_, ..., ***b***_*m*_ and modeled measurement noise **ϵ**(0, **σ**_H_2_O_). The statistical quantities β and **ϵ** can then be scaled by additional factors to simulate different levels of variability for these coefficients in a created cohort of *n* measurements. In our application, the molecular outcome ***Y*** represents an artificial measurement of a control sample and Y* represents an artificial measurement of a cancer case sample. Unless explicitly stated otherwise, the descriptive approach is used where independent experimental measurements of cases and controls form the basis of biological variability calibrations.

### Experimental FTIR Spectra and Machine Learning Analysis

The experimental FTIR spectra used in this study largely overlap with spectra obtained from a previous study that involved the detection of lung and prostate cancer from blood serum.^7^ Newly measured blood sera samples from different individuals were included in this study to increase the sample size and thus improve the statistical robustness of the results. All participants provided written informed consent for the study under research study protocol #17-141 and under research study protocol #17-182, both of which were approved by the Ethics Committee of the Ludwig-Maximillian-University (LMU) of Munich. Our study complies with all relevant ethical regulations and was conducted according to Good Clinical Practice (ICH-GCP) and the principles of the Declaration of Helsinki. The clinical trial is registered (ID DRKS00013217) at the German Clinical Trials Register (DRKS).

In total, we considered spectra from 523 lung cancer patients, 411 prostate cancer patients, and nonsymptomatic references pair-matched to each individual from each cancer entity. A detailed breakdown of the cohorts is provided in Table S1 in the Supporting Information. Information regarding the study design, statistical matching, sample collection, sample handling, FTIR measurements, and spectral preprocessing is detailed in the previous study.^7^ Machine learning analysis performed on the experimental and simulated data is detailed in Section 4 in the Supporting Information. In essence, an L2-regularized logistic regression was used as a classification algorithm. Classifications were assessed using the receiver operating characteristic (ROC) curve, and the area under the curve (AUC) was used as a summary metric of predictive performance.

## Results

By applying our simulation model to a clinically relevant scenario, we studied the potential and limitations of detecting lung and prostate cancer in binary case–control settings. Using experimentally measured FTIR spectra, we validated that comparable results were obtained between artificially generated cohorts and experimental cohorts. Next, we investigated how classification performance was influenced by varying model parameters that control the cohort size, measurement noise, biologically variability, and molecular complexity. Our findings from the lung cancer application are provided within the main text and figures. Figures relating to the prostate cancer conveyed consistent results with the lung cancer application and are thus provided in Sections 6–9 in the Supporting Information.

### Validation of Simulated Data

**Figure 2:**
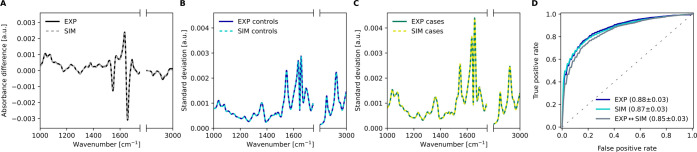
Validation of the model calibration procedure for the lung cancer detection case study. Spectral cohorts of individuals were simulated to model the properties of measured experimental cohorts using the same sample sizes (*n* = 523 cases vs 523 controls). (A) Differential fingerprint, defined as the difference between the mean of the case and control spectral features, for the experimental cohort (black) and the simulated cohorts (gray) as averaged across 10 simulation repetitions. (B, C) Standard deviation of the control spectral features (B) and case spectral features (C) for the experimental cohort (blue) and simulated cohorts (cyan) as averaged across 10 simulation repetitions. (D) ROC curve for binary case–control classifications when training and testing on experimental samples (blue), training and testing on simulated samples (cyan), and training on simulated samples and testing on experimental samples (gray). The AUCs are listed in the figure legend along with their standard deviations across the cross-validation splits. Figure S2 in the Supporting Information shows consistent results with the prostate cancer application.

After performing the calibration procedure described in the methods, we validated that the simulation model was able to create cohorts that effectively captured the intrinsic properties of experimental cohorts for our lung cancer case study (Figure 2A–D). We simulated 10 data sets of case and control samples with the same sample counts as our experimental cohorts. Each simulated cohort was created with a different set of generated random numbers (i.e., a different random seed), thereby, minimizing the effects of random perturbations. Across the 10 created data sets, we calculated the difference between the mean spectrum of case and control samples and their standard deviations across the spectrum. The resulting statistical properties were then averaged for the 10 simulated data sets and compared to the same properties of the experimental cohort.

In this comparison, we observed a high degree of agreement in the statistical features of the spectra between simulated and experimental data (Figure 2A–C). The differential fingerprint retained its structure in simulation (Figure 2A), and the standard deviations around the mean spectrum were well modeled for case and control samples (Figure 2B,C). Since our simulated cohorts were calibrated to follow the experimental cohort, it was unsurprising to find such a high degree of similarity in these properties.

Relating to our aim of studying the influence of noise factors on predictive modeling, we compared the classification performance of detecting cancer cases from controls for both experimental and simulated data (Figure 2D). An L2-regularized logistic regression classifier was used for the predictive modeling to help ensure classifier robustness against noise (Sections 4 and 11 in the Supporting Information for more details). For the experimental data, model performance was estimated in a 10-times repeated 10-fold cross-validation—totaling 100 folds. For the simulated data sets, a 10-fold cross-validation was carried out on each of the 10 simulated data sets—thereby, also totaling 100 folds. With this pipeline, we estimated mean ROC-AUCs of 0.88 and 0.87 for the lung cancer classification with the experimental data and the simulated data, respectively (Figure 2D, blue and cyan curves). The ROC-AUC values that we observed fall within the standard deviation across the cross-validation folds, validating that the degree of class separation was well modeled for our simulated data following the descriptive calibration procedure.

As an additional validation, we compared the predictive performance of a model trained exclusively on simulated data and tested on experimental data. To ensure that no experimental data used for testing contributed to the model training, we split the experimental data into 10 folds. For each fold, we held out an experimental test set, fit a predictive model on simulated samples calibrated on the remaining experimental samples, and tested the predictive model on the held-out set. As previously described, we created simulated data sets of the same cohort sizes as our experimental data and repeated the simulation 10 times, resulting in a total of 100 ROC-AUC estimates. With this approach, we still achieved a similar performance to what we expected from the experimental data estimates, with a mean ROC-AUC of 0.85 (Figure 2D, gray curve). When the machine learning model was trained on even larger sets of simulated data with *n* = 100,000 of balanced cases and controls, the experimentally obtained ROC-AUC values can be fully recovered (Figure S6 in the Supporting Information). Taken together, these results suggest that the proposed descriptive modeling approach is able to capture all relevant properties and features for the classification of the studied system.

We repeated the above numerical experiment but with a more simplistic model for generating the cancer cohort. Instead of modeling cases based on experimental measurements, we introduced a single discriminant feature vector ***d*** to describe them (eq 7 based on the experimentally obtained differential fingerprints depicted in Figure 2A). Surprisingly, the model was still able to reproduce the experimental results to a large extent (Figure S8 in the Supporting Information). Although the approach based on modeling the case measurements using experimental case spectra (Figure 2A–D) better captured the properties of the spectra, the more simplistic approach indicated that most of the relevant changes between the case and control state could be explained by a single vector.

### Influence of Cohort Size

**Figure 3:**
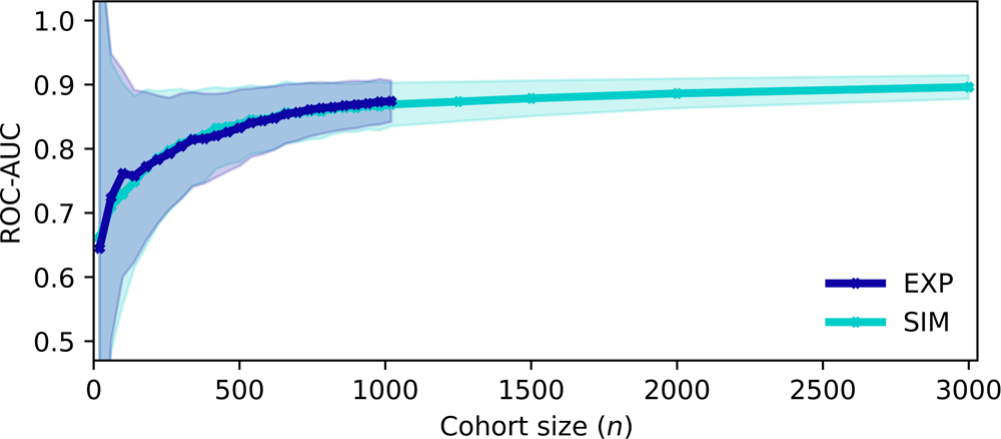
Effect of cohort size on lung cancer detection. Spectral cohorts consisting of balanced cases and controls were simulated at changing sample counts. The cross-validated ROC-AUC is plotted against the cohort size for the simulated samples (cyan). For comparison, sets of experimental samples were randomly selected and cross-validated upon to model the effects of changing cohort sizes with experimental observations (blue). The solid curves depict the average scores along with their standard deviations in the shaded region. Figure S3 in the Supporting Information shows consistent results with the prostate cancer application.

The simulation approach allows for generating artificial cohorts in arbitrary counts, which enabled us to carry out an investigation on the influence of cohort size on predictive performance. We used the previously calibrated parameters of the simulation model and tested out different cohort sizes (Figure 3). For each cohort size tested, 100 artificial cohorts were generated and the ROC-AUC was again determined in 10-fold cross-validation on each cohort size. In addition, a similar investigation was carried out using the experimental observations, where samples were randomly selected in 100 different iterations, selecting a different set of measurements in each iteration for each cohort size, and cross-validated upon (10-fold).

We observed a similar dependence between the cohort size and the performance of detecting lung cancer for simulated and experimental data (Figure 3). This investigation revealed that increasing the size of the simulated data sets beyond the number of experimentally available data (*n* = 1046) only marginally improved the classification performance and reduced the relative standard deviation as the plateau of asymptotic performance was reached. It was apparent, however, that the classification performance significantly suffered when smaller cohorts were used (e.g., *n* ≤ 200) compared to using larger cohorts (e.g., with *n* ≥ 1000). Moreover, the standard deviation became intolerably large with smaller sample sizes, making it difficult to construct conclusive estimates when only limited cohort sizes are available.

This result showed that the cohort sizes used were sufficiently large, which is reflected by the well-known fact that the predictive performance of a machine learning classifier reaches a plateau above a certain cohort size.^35,36^ Furthermore, it served as an additional validation that the simulation model was able to reproduce the results obtained from experimental observations.

### Influence of Biological Variability and Measurement Noise

**Figure 4:**
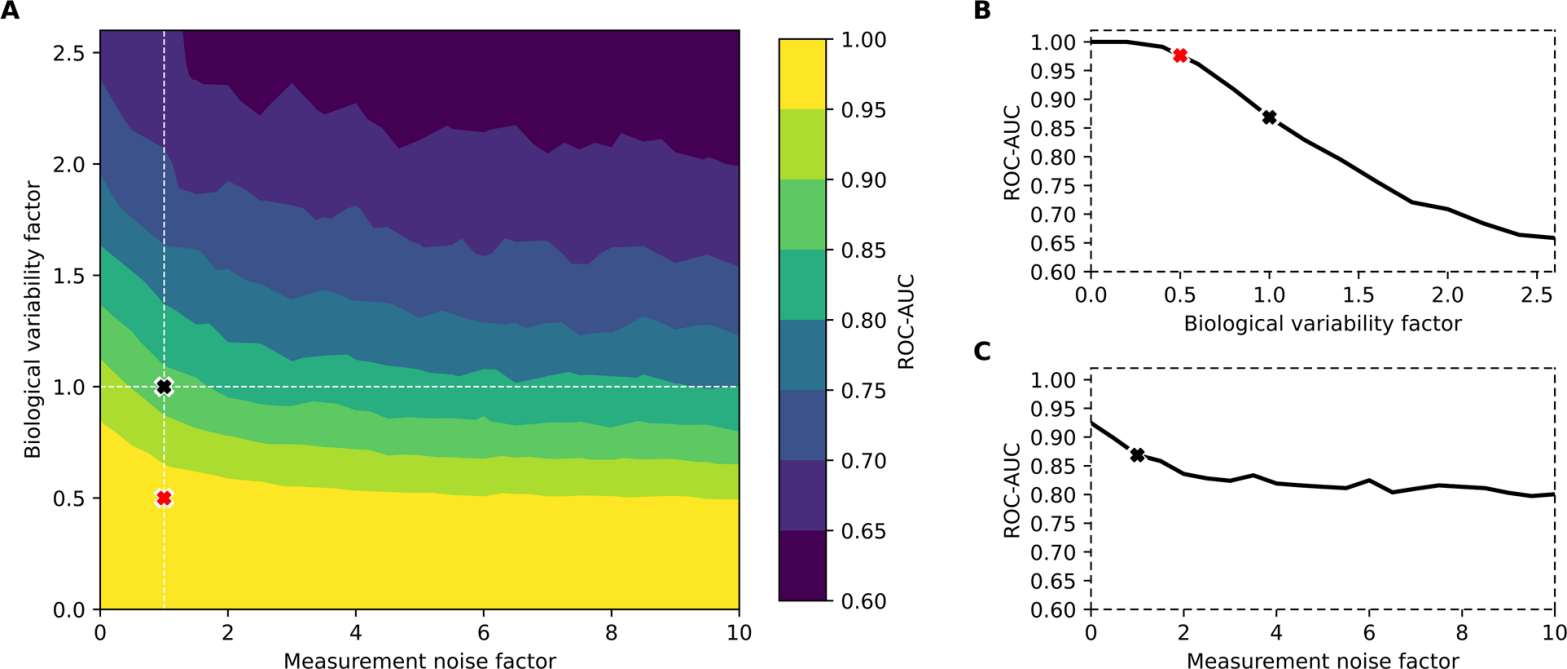
Effect of biological variability and measurement noise on lung cancer detection. (A) Classification performance was measured by the mean cross-validated ROC-AUC on multiple spectral cohorts simulated at changing levels of biological variability and measurement noise. The black “***x***” marks the point of calibration where the simulated cohorts model the experimental levels of between-person biological variability and measurement noise. The red “***x***” marks an estimate for reduced biological variability in a longitudinal, self-referencing, scenario as estimated from previous work.^14^ (B) Slice of (A) when the measurement noise factor is held constant at the calibrated level and the biological variability factor is changing. (C) Slice of (A) when the biological variability factor is held constant at the calibrated level and the measurement noise factor is changing. Figure S4 in the Supporting Information shows consistent results with the prostate cancer application.

After demonstrating that the calibration procedure generated artificial cohorts that captured the properties of our experimental results, we examined the potential effects of measurement noise and biological variability on the performance of detecting lung cancer (Figure 4). When describing the simulation model, we considered the random coefficients β and **ϵ**, modeling the biological variability and measurement noise in levels similar to that of the experimental observations. The standard deviations of these two coefficients are tunable, thus enabling the scaling of these levels noise factors seen within the spectra and thereby simulating different experimental conditions. We generated artificial cohorts considering varying levels of these noise factors and examined their effects on classification performance. For each level of measurement noise and biological variability considered, we simulated 10 cohorts and cross-validated (10-fold) on each simulated cohort—averaging the ROC-AUC scores across the 100 total test data splits.

Overall, we found that the dominating source of noise, which masked away the signal separating the classes, was the level of biological variability (Figure 4A). With no added biological variability, the binary classifiers were able to distinguish the clear signal of the diseases, achieving near-perfect to perfect ROC-AUCs within the explored levels of measurement noise. With increasing levels of biological noise, the disease signals were increasingly masked away, leading to sharp declines in ROC-AUCs down to the plateau of observing random chance models. Our point of calibration fell within a region where minor changes to the biological variability have significant effects on the performance of distinguishing cases from controls (Figure 4B).

Previously, it was found that the level of within-person biological variability in molecular fingerprints over a period of 6 months was near a factor of 2 less, on average, than the level of between-person variability of individuals in groups.^14^ This allowed us to estimate the potential classification performance of detecting cancer in a personalized, longitudinal health-monitoring scenario. By tuning our random coefficient β to model the level of within-person variability (i.e., β × 0.5), we estimated that such a classification would yield a ROC-AUC of 0.98 for lung cancer detection (Figure 4A,B).

Compared to the effect of biological variability, varying the level of measurement noise was found to have a substantially smaller effect on the classification performance (Figure 4A,C). Eliminating the modeled measurement noise (i.e., **ϵ** × 0) yielded an estimated ROC-AUC of 0.92. Nevertheless, varying both the biological variability and measurement noise simultaneously revealed that the effect of the measurement noise on the retrieved ROC-AUC also depended on the level of biological variability. For instance, when the level of biological variability was highest, reducing the measurement noise resulted in substantial classification performance gains (in terms of ΔROC-AUC). However, when the biological variability was lowest, measurement noise had little effect on the retrieved ROC-AUC—with the classification performing with perfect efficiency. Thus, advances in spectroscopic methods which result in further reductions of measurement noise may be particularly promising for problems with low classification efficiencies (e.g., due to weak disease signatures).

### Influence of Molecular Complexity

Within the proposed simulation model, the biological variability of the molecularly complex blood-based spectra was modeled according to two aspects: the total strength of biological variability, determined by the statistical variable β, and the structure of the underlying biological variability as determined by the set of *m* unique and independent calibration vectors ***b***_*i*_ of a given sample pool. Each vector represents the absorbance spectrum combining all molecular constituents in a given molecular sample, combining all their respective concentrations. As previously described, changing the number of independent vectors considered when creating an artificial sample would mathematically correspond to changing the number of unique molecular species creating the full blood-serum spectrum.

**Figure 5:**
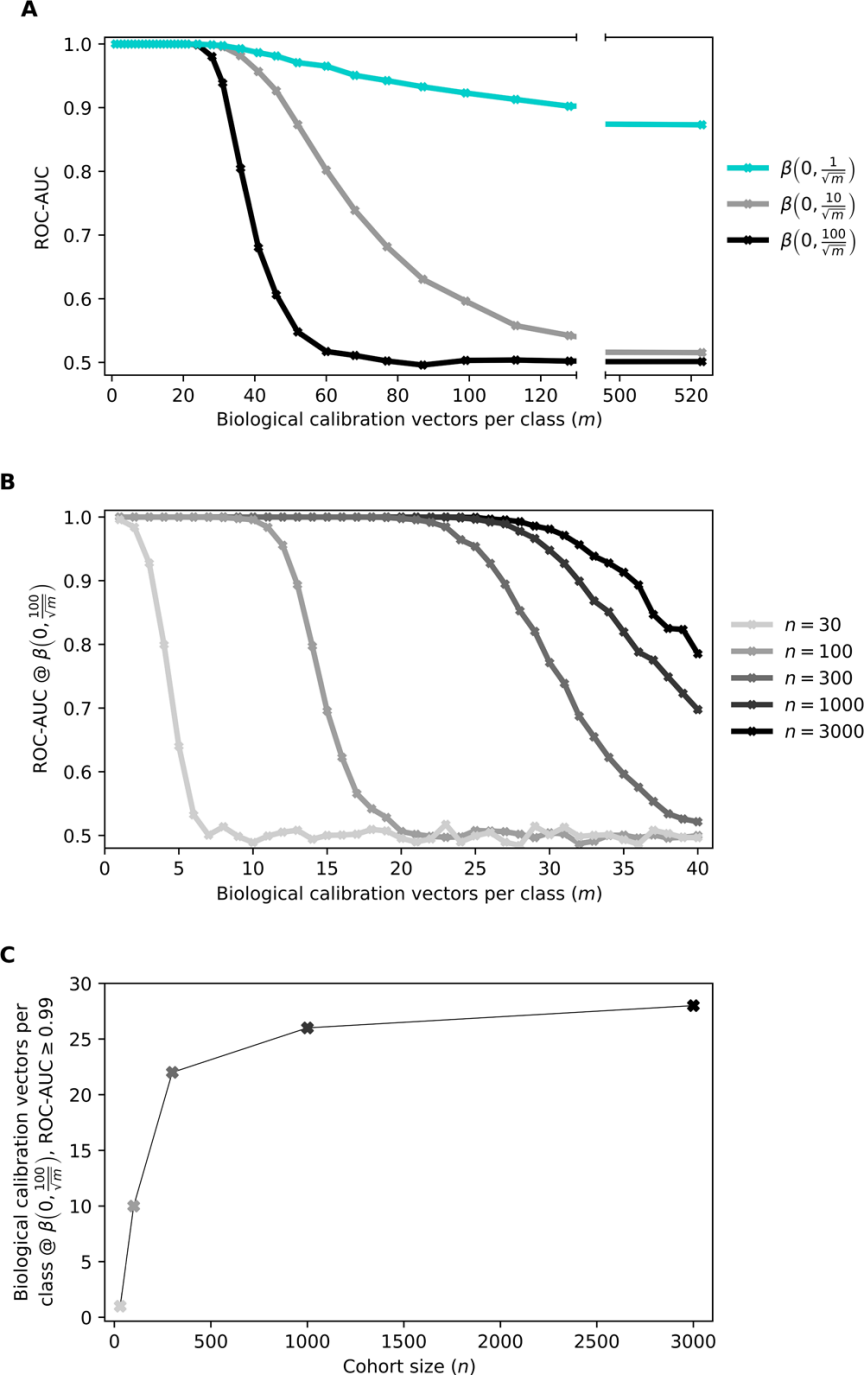
Influence of molecular complexity on lung cancer detection. (A) Multiple spectral cohorts were simulated at changing levels of *m* (i.e., the number of calibration vectors defining each molecular state) and changing levels of biological variability (β). Cohorts were created with the same cohort size as the experimental measurements and cross-validated upon to estimate the ROC-AUC as a measure of prediction performance. (B) Multiple data sets were simulated at changing levels of *m* and changing cohort sizes (*n*) to investigate the effects of the sample size on the trends observed in (A) at the highest level of biological variability investigated. (C) Number of calibration vectors *m* required to achieve a ROC-AUC ≥ 0.99 at the cohort sizes investigated in (B). Figure S5 in the Supporting Information shows consistent results with the prostate cancer application.

To model this, we kept the mean measurement ***b*®** for each sample pool constant and randomly selected *m* different experimental calibration vectors ***b***_*i*_ to create artificial cohorts at varying values of *m* (Figure 5A–C). Since ***b*®** is held constant, we assume that altering the molecular complexity has no direct effect on the disease signal, but in fact, increasing complexity serves to mask it away. To note as well, the case and control cohort spectra are each based on a separate set of vectors, and thus, the entire set of case and control spectra is based on 2 × *m* independent vectors.

In the first numerical experiment, we kept the relative strength of biological variability at the same level as previously calibrated, utilizing the random coefficient . The measurement noise coefficient **ϵ** was also kept at the calibrated level when creating the simulated samples. Cohorts for lung cancer were created with the same sample sizes as our experimental measurements. This process was repeated 100 times, each with a different set of *m* randomly selected experimental measurements, and cross-validated (10-fold) on each simulated data set. We observed that when the molecular complexity was modeled with *m* ≤ 20 independent biological calibration vectors for each sample pool, perfect class separation can be achieved with ROC-AUCs of 1.0 (Figure 5A, cyan curve). Increasing the molecular complexity of the spectra past this, using calibration vectors that did not contribute to the cancer disease signal, added a level of molecular complexity that reduced the prediction confidence of the classification models.

This observation prompted the question of whether there might be a more general relationship between molecular complexity, biological variability, and the ability of infrared fingerprinting to distinguish between two classes. Therefore, we repeated the previous experiment, but with larger levels of variability for β, representing the strength of the biological variability. We found that perfect separation was possible for *m* ≤ 20, irrespective of the level of biological variability in our explored domain (Figure 5A, gray and black curves). From a mathematical point of view, this suggests that at such a threshold, the underlying system of equations to be solved is over-determined and thus has a unique solution that a predictive model is able to find.

Further investigations revealed that the threshold value for *m*, for which perfect separation is still possible, can be increased with increasing cohort size but only at an exponentially decreasing rate (Figure 5B,C). In other words, more individual components could be detected at a higher level of sensitivity by measuring exponentially more samples. However, this threshold certainly depends on the correlation of the calibration vectors, the spectral bandwidth, and resolution, and can vary depending on the application.

We would like to note that the threshold values obtained here can only be transferable under idealistic conditions. In particular, the application of interest must not be tainted by any measurement noise, and the signal of interest, as well as all other molecules, must be of similar concentration. Nevertheless, these results clearly reflect that the capacity of infrared molecular fingerprinting of complex samples can be drastically increased—if the number of components of the analyzed matrix in which the spectral marker is embedded is significantly reduced below 100.

## Discussion

Capturing biological phenotypes through molecular fingerprinting forms the basis for many innovative applications, such as disease detection. Nevertheless, the full potential and limitations of these approaches are not fully understood due to the complex interplay of measurement noise, biological variability, and the actual distinctive molecular pattern.

To investigate these effects, we systematically adjusted the parameters of our validated *in silico* model. We found that improved classification was achievable by decreasing the levels of biological variability and measurement noise. Reducing measurement noise promises to improve classification efficiency by a few percentage points (in terms of ROC-AUC scores) and can potentially be exploited with next-generation infrared spectrometers.^37^ A greater advantage came from reducing biological variability, which is more challenging to realize as it is inherent to any biological setting—from a single gene^38^ to an organismal level.^12,13^ A possible strategy here is populational stratification, or grouping individuals into defined strata (e.g., by age, body mass index, and lifestyle factors), to reduce the biological variability within each stratum. Although widely established in genomics,^39^ it needs to be carefully controlled to not lead to spurious associations. Furthermore, the concept of longitudinal self-referencing could be incorporated in fingerprinting for disease diagnostics and health monitoring.^40^ The within-person biological variability of blood-based infrared fingerprints is near a factor of 2 lower than the between-person variability when monitored over a span of 6 months.^14^ Using our model, we estimated that this would improve the ROC-AUC from 0.88 for group comparison to 0.98 for longitudinal self-referencing when detecting lung cancer.

Further investigations showed that identifying spectral patterns using infrared molecular fingerprinting may be fundamentally limited by the molecular complexity of the analyzed samples. By reducing the number of different molecular species considered for generating the cohorts, perfect class separation was achievable when less than 20 unique spectra were used for each sample pool—regardless of the level of biological variability. By increasing the cohort size, near-perfect separation could be achieved even with increasing molecular complexity—albeit, this requires exponentially larger cohort sizes. This indicated that infrared fingerprinting of complex biological samples (e.g., blood-based media) is only able to unambiguously determine a certain number of independent variables, or molecular species, within complex samples. The actual number of detectable independent variables certainly depends on measurement properties such as the spectral bandwidth, spectral range, and the type of sample under investigation. Such questions can be investigated with the presented model but would exceed the scope of this study. Considering our applications using blood-serum, we found that reducing the chemical sample complexity (e.g., via chromatography) could be a viable option to improve the classification performance.

Critically seen, we initially developed our approach to calculate realistic fingerprint spectra of blood serum by considering the individual spectral contributions of the majority of molecular species contained therein. Since this requires information on individual molecular concentrations in blood for different groups of the human population and also the use of a large number of individual component spectra, with the majority unavailable, we could not realize this approach. Instead, we chose a descriptive approach that relies on measured spectra of the molecularly complex samples from observational studies. We have, however, shown that both approaches follow a similar mathematical formulation. To assess whether they have similar explanatory power, a direct comparison is necessary and which is already ongoing. At the same time, we believe that the bottom-up approach can facilitate the analysis of simpler systems, such as pharmaceutical samples,^33^ to investigate the effects of factors like molecular mixing and variance of molecular concentrations introduced through manufacturing.

A disadvantage of the descriptive approach is that the best agreement with experimental results is only achieved when healthy and diseased sample pools were calibrated separately. Choosing a simplified approach that describes the occurrence of a disease by a single discriminant vector, but otherwise only considers the biological variability of the healthy cohort, reduces the agreement with the experimental results (Figure S8 in the Supporting Information). Nevertheless, this approach has its advantages. It is well suited for exploratory analysis of any fingerprinting settings and could help estimate to what extent a change in a certain molecule (e.g., a studied biomarker) would be detectable by fingerprinting. For such investigations, one would only need to record the spectra of the target molecule independently and then use the calibrated data from this work to generate simulated spectra with and without contributions from the target spectra.

In general, the core idea of the proposed *in silico* model can be transferred to other molecular fingerprinting techniques, such as Raman spectroscopy^20^ or nuclear magnetic resonance spectroscopy,^21^ and to other applications involving multiclass classifications. Furthermore, the model can be applied to study the proper applications of machine learning algorithms—from investigating the effects of noise on hyperparameter tuning (Section 11 in the Supporting Information) to how different classification algorithms perform under different simulated conditions. To facilitate the use of the model, we provide a Python toolbox alongside executable scripts to reproduce and extend the results of this study (Sections 2 and 3 in the Supporting Information).

## Conclusions

In this study, we describe an *in silico* approach capable of modeling molecular fingerprints of complex biological systems. We calibrated our model using experimentally measured infrared spectra of blood sera and applied it to the detection of lung and prostate cancer. Excellent agreement between the statistical properties and classification results of simulated and experimental data was achieved for both applications. The validity of the approach was further supported by the finding that machine learning models trained on simulated data and tested on experimentally measured data delivered comparable results. We demonstrated that the newly developed model enables investigations of the potential and limitations of the molecular fingerprinting framework and that it can serve as a platform for exposing opportunities to advance molecular fingerprinting applications.

## Data Availability

Datasets and Python scripts: https://github.com/tarek-eissa/in-silico-spectral-generator.

## Supporting Information Available

Experimental cohort description, data and code availability, additional methodological details, and supplementary figures (PDF)
Datasets and Python scripts (ZIP)
