# Supplementary material for: Limits and Prospects of Molecular Fingerprinting for Phenotyping Biological Systems Revealed through In Silico Modeling
Source: Anal Chem. 2023 Apr 12;95(16):6523–32. doi: 10.1021/acs.analchem.2c04711 (PMC10134135; doi:10.1021/acs.analchem.2c04711)
Supplement: Supplementary file 1 — ac2c04711_si_001.pdf [file ac2c04711_si_001.pdf]

Supporting information for:
"Limits and prospects of molecular fingerprinting for phenotyping biological systems revealed through *in silico* modeling"

Tarek Eissa[1,3], Kosmas V. Kepesidis[1,2], Mihaela Zigman[1,2,*], and Marinus Huber[1,2,*]

[1] Ludwig Maximilian University of Munich (LMU), Department of Laser Physics, 85748 Garching, Germany
[2] Max Planck Institute of Quantum Optics (MPQ), Laboratory for Attosecond Physics, 85748 Garching, Germany
[3] Technical University of Munich (TUM), Department of Informatics, 85748 Garching, Germany
[*] Correspondence: mihaela.zigman@mpq.mpg.de, marinus.huber@mpq.mpg.de

Contents

1 Description of experimental cohort S-2
2 Analysis code S-2
3 Data access S-2
4 Machine learning analysis S-2
5 Modeled FTIR measurement noise S-3
6 Validation of simulated data for the prostate cancer application S-3
7 Influence of cohort size for the prostate cancer application S-4
8 Influence of biological variability and measurement noise for the prostate cancer application ... S-4
9 Influence of molecular complexity for the prostate cancer application S-5
10 Simulating larger cohorts for model validation S-6
11 Influence of noise on predictive model tuning S-7
12 Simplified *in silico* model using a single vector to model case measurements S-8
References S-8

1 Description of experimental cohort

Table S1 provides sample size, age, sex, and body mass index (BMI) descriptions of the experimental lung and prostate cancer cohorts. These cohorts were used for both - calibrating the biological variability of the simulated samples and for validating the statistical properties of the simulated cohorts. Details about the study design, sample collection and handling, and measurements of the samples are described in a previously published work.[1]

	Lung cancer cohort		Prostate cancer cohort	
	Cases	Controls	Cases	Controls
n	523	523	411	411
% male	54%	54%	100%	100%
Age (mean ± SD)	67.2 ± 9.7 years	64.2 ± 10.4 years	65.2 ± 9.1 years	59.2 ± 15.9 years
BMI (mean ± SD)	25.7 ± 4.9 kg/m^2	26.4 ± 5.0 kg/m^2	27.1 ± 4.0 kg/m^2	26.7 ± 4.9 kg/m^2

Table S1: Description of the experimental lung cancer and prostate cancer cohorts. For each entity, cancer cases were pair-matched to non-symptomatic controls based on sex, age, and BMI.

2 Analysis code

To facilitate the use of the model, the Python code used for generating the artificial spectra is deposited in a GitHub repository.[2] There, we further provide Python scripts (on the Jupyter Notebook framework) to perform and reproduce all simulations shown in the results of the main text of the article.

3 Data access

Making use of the proposed model, we provide a dataset of generated lung and prostate cancer cohorts of FTIR spectra which model the data used within this work (n = 100000 for each cancer entity) in a GitHub repository.[2] The dataset was created using our experimental blood serum measurements for each cancer entity and the matched non-symptomatic controls. The artificial cohorts were created by following the described procedure for calibrating the biological variability using experimental measurements and the measurement noise using repeated water measurements. The water measurements (n = 400) used for calibrating the measurement noise are also deposited, along with the generated artificial cohorts. This data can serve as an input for generating additional artificial cohorts for *in silico* investigations, as well as be employed as a basis for gaining insights when limited numbers of serum-based measurements are at hand.

4 Machine learning analysis

Classification performance was evaluated by calculating the area under the receiver operating characteristic curve (ROC-AUC) of trained L2-penalized logistic regressions. To estimate the ROC-AUC score on unseen test samples, the data was first split according to a 10-fold stratified cross-validation. On the training splits of each fold, an exhaustive grid search was carried out using an inner 3-fold stratified cross-validation to determine an optimal value for the cost parameter C of the logistic regression[3] – the value which maximizes the ROC-AUC estimated on the inner validation sets. Once an optimal value was determined, the logistic regression was re-fit on all samples of a given training split and tested on the corresponding outer set of testing samples to provide an unbiased estimate of performance on unseen data. The outer 10-fold cross-validation was repeated for some problems and was mentioned in the corresponding section of the results. The ROC-AUC scores across all folds were averaged and reported along with their standard deviation. When investigating the effect of the cost parameter C (Section 11 in the Supporting Information), the inner cross-validation and grid search steps were skipped and the results with all C values were shown. Data standardization – done by subtracting the mean and dividing by the standard deviation of each spectral feature – was applied as a preprocessing step before training and testing the logistic regression models. All mean and standard deviation values were calculated from the training splits and applied to their corresponding testing splits. The logistic regression, cross-validation, and grid search algorithms were used as implemented in the Scikit-Learn open-source Python package (version 0.24.1).[4]

5 Modeled FTIR measurement noise

To determine a realistic estimate for the measurement noise of the FTIR measurement device, a total of 400 water samples were repeatedly measured with the FTIR spectrometer. The standard deviation between the performed measurements across the spectral features was subsequently calculated. Fig. S1**A** depicts water measurements performed on the FTIR spectrometer. These measurements were used to calibrate the additive measurement noise introduced into the model (illustrated in Fig. S1**B**).

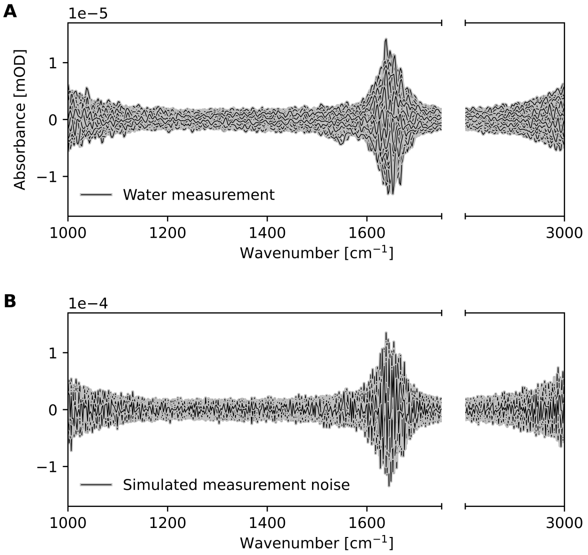

Figure S1: Calibration of the measurement noise using repeated water FTIR measurements.

(**A**) Water reference absorbance spectra (n = 400) with the mean of all water measurements subtracted. (**B**) A set of random vectors generated based on the standard deviation of the repeated water measurements across the wavenumbers on an arbitrary y-axis. The vectors in (**B**) illustrate the calibrated measurement noise coefficient $\boldsymbol{\epsilon}$ in the simulation model and were scaled by a factor of 9.15 to account for the L2 vector normalization in the preprocessing of the sera measurements.

6 Validation of simulated data for the prostate cancer application

Fig. S2 supplements Fig. 2 in the main text which validated the model calibration procedure. Here, the validation was applied to the prostate cancer case study.

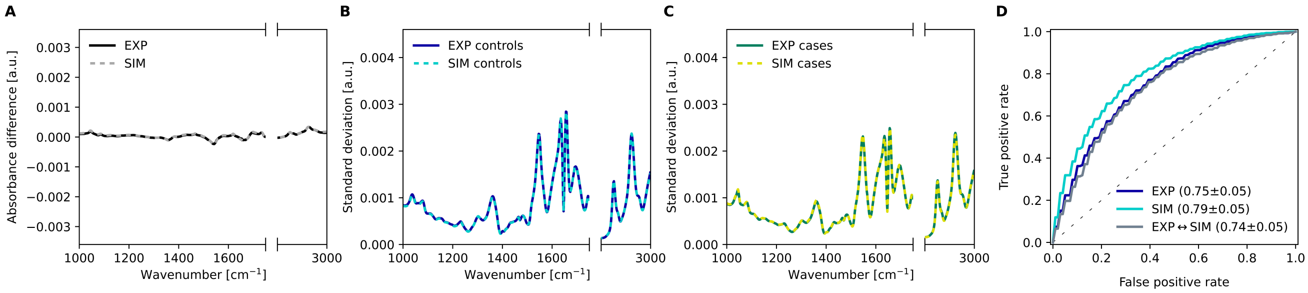

Figure S2: Validation of the model calibration procedure for the prostate cancer case study.

Spectral cohorts of individuals were simulated to model the properties of measured experimental cohorts using the same sample sizes (n = 411 cases vs. 411 controls). (**A**) The differential fingerprint, defined as the difference between the mean of case and control spectral features, for the experimental cohort (black) and the simulated cohorts (gray) as averaged across 10 simulation repetitions. (**B**-**C**) The standard deviation of the control spectral features (**B**) and case spectral features (**C**) for the experimental cohort (blue) and simulated cohorts (cyan) as averaged across 10 simulation repetitions. (**D**) The ROC curve for binary case-control classifications when training and testing on experimental samples (blue), training and testing on simulated samples (cyan), and training on simulated samples and testing on experimental samples (gray). The AUCs are listed in the figure legend along with their standard deviations across the cross-validation splits.

7 Influence of cohort size for the prostate cancer application

Fig. S3 supplements Fig. 3 in the main text which investigated the effect of the cohort size on classifier performance for experimental and simulated cohorts. Here, the validation was applied to the prostate cancer case study.

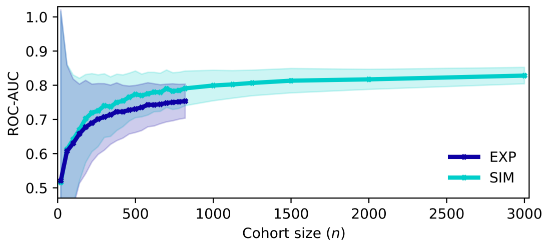

Figure S3: Effect of cohort size on prostate cancer detection.

Spectral cohorts consisting of balanced case and control samples were simulated at changing sample counts. The cross-validated ROC-AUC is plotted against the cohort size for the simulated samples (cyan). For comparison, sets of experimental samples were randomly selected and cross-validated upon to model the effects of changing cohort sizes with the experimental observations (blue). The solid curves depict the averaged scores along with their standard deviations in the shaded region.

8 Influence of biological variability and measurement noise for the prostate cancer application

Fig. S4 supplements Fig. 4 in the main text which investigated the effects of measurement noise and biological variability on classifier performance. Here, the validation was applied to the prostate cancer case study.

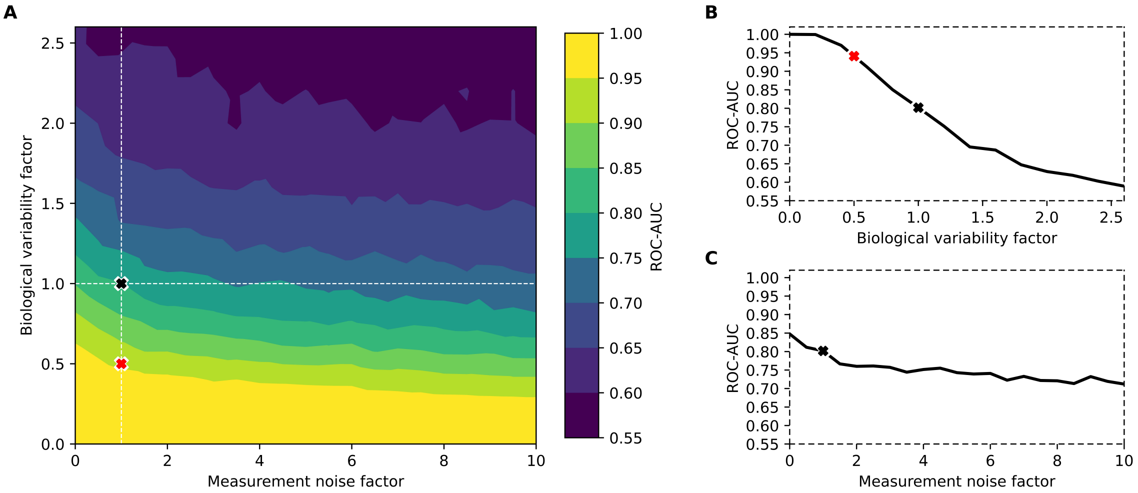

Figure S4: Effect of biological variability and measurement noise on prostate cancer detection.

(**A**) Classification performance was measured by the mean cross-validated ROC-AUC on multiple spectral cohorts simulated at changing levels of biological variability and measurement noise. The black "**x**" marks the point of calibration where the simulated cohorts model the experimental levels of between-person biological variability and measurement noise. The red "**x**" marks an estimate for reduced biological variability in a longitudinal, self-referencing, scenario as estimated from previous work.[5] (**B**) Depicts a slice of (**A**) when the measurement noise factor is held constant at the calibrated level and the biological variability factor is changing. (**C**) Depicts a slice of (**A**) when the biological variability factor is held constant at the calibrated level and the measurement noise factor is changing.

9 Influence of molecular complexity for the prostate cancer application

Fig. S5 supplements Fig. 5 in the main text which investigated the effect of chemical molecular complexity on classifier performance. Here, the validation was applied to the prostate cancer case study.

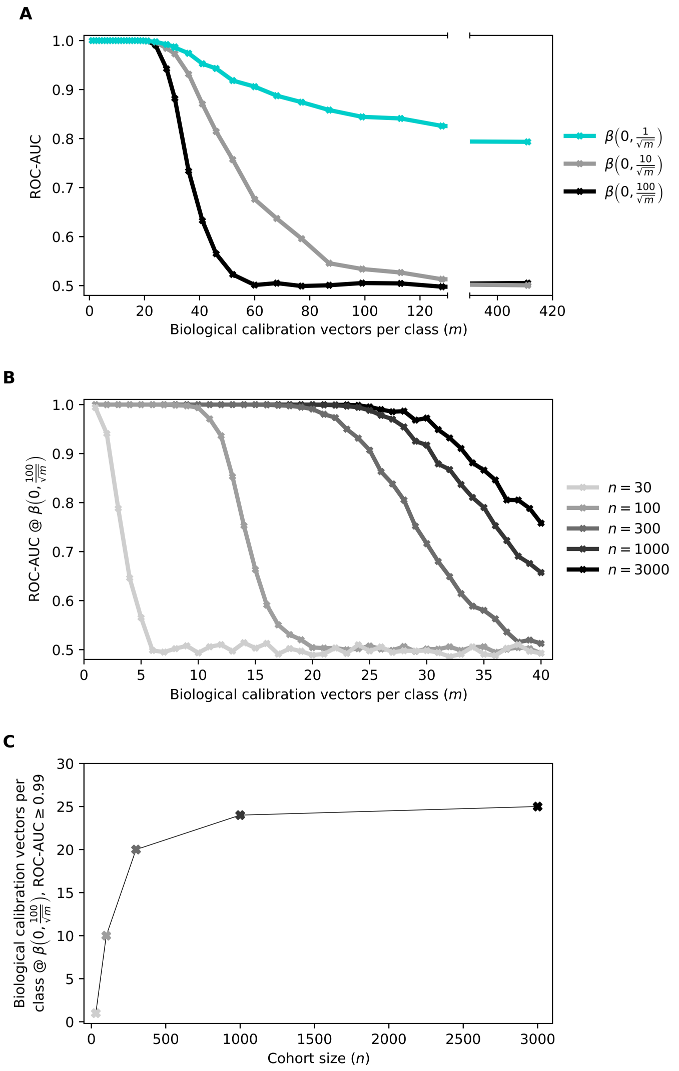

Figure S5: Influence of molecular complexity on prostate cancer detection.

(**A**) Multiple spectral cohorts were simulated at changing levels of *m* (i.e., the number of calibration vectors defining each molecular state) and changing levels of biological variability (β). Cohorts were created with the same cohort size as the experimental measurements and cross-validated upon to estimate the ROC-AUC as a measure of prediction performance. (**B**) Multiple datasets were simulated at changing levels of *m* and changing cohort sizes (*n*) to investigate the effects of the sample size on the trends observed in (**A**) at the highest level of biological variability investigated. (**C**) The number of calibration vectors *m* required to achieve a ROC-AUC $\geq$ 0.99 at the cohort sizes investigated in (**B**).

10 Simulating larger cohorts for model validation

Fig. S6 supplements Fig. 2**D** in the main text and S2**D**. Here, larger simulated cohorts were created to validate a model trained on simulated data and tested on experimental data. In comparison to Fig. 2**D** and S2**D**, the ROC-AUC values could be fully recovered using the larger sample sizes for both the lung (Fig. S6**A**) and prostate cancer (Fig. S6**B**) applications.

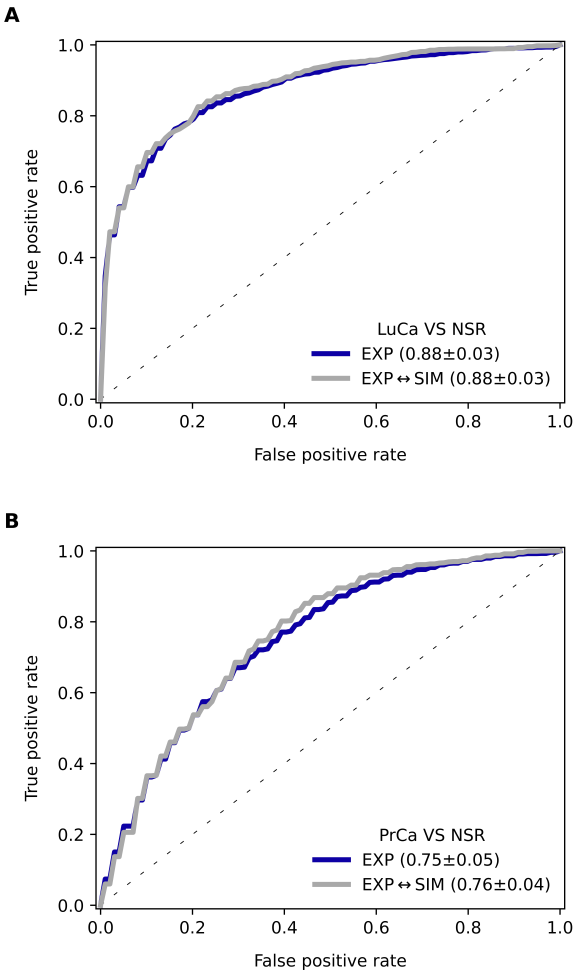

Figure S6: Receiver operating characteristic curves for binary classifications when trained on $n = 100000$ simulated samples of balanced classes and tested on unseen sets of experimental samples in a cross-validation (in gray). The blue curve depicts the result when cross-validating on the experimental samples. The areas under the curves and their standard deviations are listed in the figure legends for the lung cancer (**A**) and prostate cancer (**B**) applications.

11 Influence of noise on predictive model tuning

Regularization plays a pivotal role in the predictive modeling of ill-posed problems such as the classifications explored within this study. Techniques of regularization help predictive models mitigate the effects of multicollinearity and noise present in the training data.[6,7] By introducing an L2 penalty, a supervised machine learning model works to solve the following generalized optimization problem, given a set of instance-label pairs $(\boldsymbol{x}_i, y_i)$:

$$\min_{\boldsymbol{w}} \quad \frac{1}{2}\boldsymbol{w}^T\boldsymbol{w} + C\sum_{i=1}^{N} \xi(\boldsymbol{w}; \boldsymbol{x}_i, y_i)$$

where $\xi(\boldsymbol{w}; \boldsymbol{x}_i, y_i)$ is a defined error, or loss, function used in a predictive model. In other words, the optimization works to find the weight vector $\boldsymbol{w}$ that minimizes the error between predicted outcomes and the known outcomes. To avoid a solution where $\boldsymbol{w}$ becomes unduly large in magnitude, overfitting to peculiarities in the training data, the L2 penalty term is introduced and the extent of its involvements is controlled by the free parameter $C > 0$. The closer the value of C is to 0, the sparser the solution – where the entries of $\boldsymbol{w}$ are closer to 0. The optimal value for C can be determined by cross-validating to find the value which strikes the balance between allowing the model to learn the best possible value for $\boldsymbol{w}$ and not overfit.

We used our simulation model to investigate the effects of regularization on the performance of detecting lung and prostate cancer using L2-regularized logistic regression models at varying measurement noise and biological variability levels (Fig. S7**A**-**D**). We simulated artificial cohorts at increments of increasing measurement noise, holding the biological variability at the calibrated level, and subsequently cross-validated on each simulated dataset with different values for the parameter C. A similar evaluation was repeated to examine the effect of increasing biological variability while keeping the measurement noise at the calibrated level.

We found that with heavy classifier regularization (e.g., $C = 2^{-12}$), the classification performance on unseen data samples was relatively robust to increasing levels of measurement noise (Fig. S7**A** and **C**). Such heavily regularized models, however, did not provide optimal classification performance at the explored levels of measurement noise and led to "underfit" models that failed to capture the complexity of the problem. With little-to-no measurement noise, classifiers with weaker regularization strengths (e.g., $C = 2^{12}$) consistently outperformed in testing performance. Since such weakly regularized models are very susceptible to overfitting, increasing measurement noise quickly led to the loss of generalization and the classifier under-performed on the testing sets while perfectly separating the training samples.

Comparatively, the optimal regularization parameter was less sensitive to varying levels of biological variability (Fig. S7**B** and **D**). The optimal regularization strength ($C = 2^6$), remained optimal in the explored biological variability domain. Moreover, for this type of noise, all explored values for C suffered in a similar way from increasing noise. This revealed that the optimal penalty does not depend on the biological variability and thus the effects of increased biological variability cannot be mitigated by tuning a better classifier.

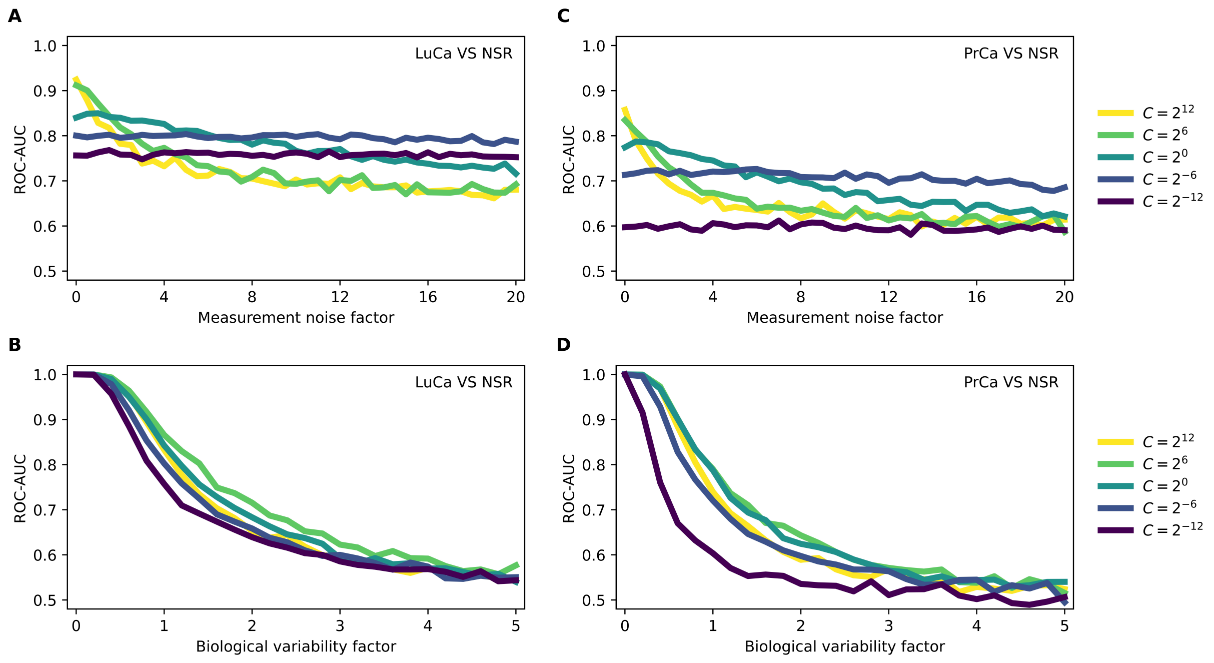

Figure S7: Influence of measurement noise and biological variability on classifier regularization.

Binary classifiers, with changing regularization strengths (parameter C), were fit to detect cancer on spectral cohorts simulated at different levels of measurement noise (first row) and biological variability (second row). (**A**-**B**) Depict the effect on detecting lung cancer while (**C**-**D**) depict the effect on detecting prostate cancer. Performance, as measured by the ROC-AUC, was estimated by cross-validating and averaging the score on each cohort created.

12 Simplified *in silico* model using a single vector to model case measurements

Fig. S8 repeats the validation of the numerical simulation shown in Fig. 2 in the main text and supporting Fig. S2, but by introducing a single discriminant feature vector $\boldsymbol{d}$ to describe the case measurements and only considering the biological variability of the non-symptomatic references to calibrate the model (equation 7 in the main text).

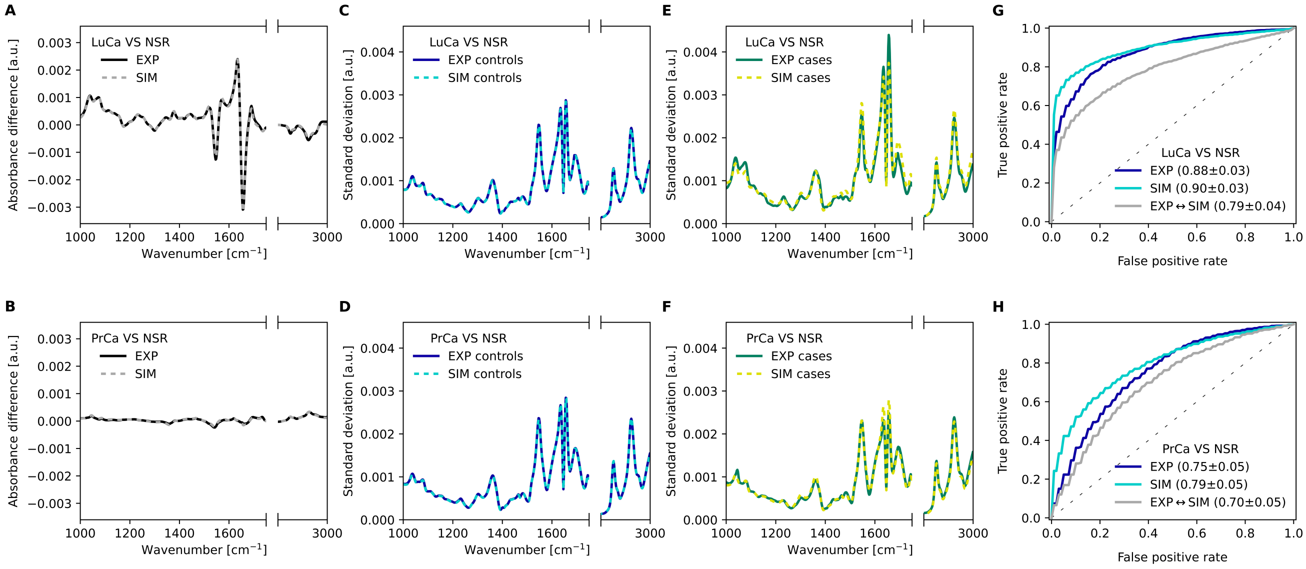

Figure S8: Validation of the calibration procedure for the model variant that uses a single discriminant vector to distinguish between cases and controls. In this investigation, the biological variability was calibrated based on the control measurements for each cancer cohort. (**A**-**B**) The differential fingerprint, defined as the difference between the mean of the case and control spectral features, for the experimental cohort and the simulated cohorts as averaged across 10 simulation repetitions. This differential fingerprint was introduced to each simulated case measurement to model the difference between the classes using a single vector for the lung (**A**) and prostate (**B**) cancer applications. (**C**-**D**) The standard deviation of the control spectral features for the experimental cohort and simulated cohorts as averaged across 10 simulation repetitions for both lung cancer (**C**) and prostate cancer (**D**) applications. (**E**-**F**) The standard deviation of the case spectral features for the experimental cohort and simulated cohorts was averaged across 10 simulation repetitions for both lung cancer (**E**) and prostate cancer (**F**) applications. For the simulated case measurements (**E**-**F**), the biological variability was scaled to minimize the squared error between the simulated standard deviation spectrum and the experimentally derived one. (**G**-**H**) The receiver operating characteristic curves (ROCs) for binary case-control classifications of lung cancer (**G**) and prostate cancer (**H**) when training and testing on experimental samples (blue), training and testing on simulated samples (cyan), and training on simulated samples and testing on experimental samples (gray). The areas under the curves are listed in the figure legends along with their standard deviations across the cross-validation splits.

References

(1) Huber, M.; Kepesidis, K. v; Voronina, L.; Fleischmann, F.; Fill, E.; Hermann, J.; Koch, I.; Milger-Kneidinger, K.; Kolben, T.; Schulz, G. B.; Jokisch, F.; Behr, J.; Harbeck, N.; Reiser, M.; Stief, C.; Krausz, F.; Zigman, M. Infrared Molecular Fingerprinting of Blood-Based Liquid Biopsies for the Detection of Cancer. *Elife* **2021**, *10*. https://doi.org/10.7554/eLife.68758.

(2) Eissa, T. In Silico Spectral Generator. *GitHub repository*. GitHub 2022. https://github.com/tarek-eissa/in-silico-spectral-generator.

(3) Fan, R. E.; Chang, K. W.; Hsieh, C. J.; Wang, X. R.; Lin, C. J. LIBLINEAR: A Library for Large Linear Classification. *Journal of Machine Learning Research* **2008**, *9*.

(4) Pedregosa, F.; Varoquaux, G.; Gramfort, A.; Michel, V.; Thirion, B.; Grisel, O.; Blondel, M.; Prettenhofer, P.; Weiss, R.; Dubourg, V.; Vanderplas, J.; Passos, A.; Cournapeau, D.; Brucher, M.; Perrot, M.; Duchesnay, É. Scikit-Learn: Machine Learning in Python. *Journal of Machine Learning Research* **2011**, *12*.

(5) Huber, M.; Kepesidis, K. v; Voronina, L.; Božić, M.; Trubetskov, M.; Harbeck, N.; Krausz, F.; Žigman, M. Stability of Person-Specific Blood-Based Infrared Molecular Fingerprints Opens up Prospects for Health Monitoring. *Nat Commun* **2021**, *12* (1)https://doi.org/10.1038/s41467-021-21668-5.

(6) Bauer, F.; Lukas, M. A. Comparing Parameter Choice Methods for Regularization of Ill-Posed Problems. *Math Comput Simul* **2011**, *81* (9), 1795–1841. https://doi.org/10.1016/j.matcom.2011.01.016.

(7) Zou, H.; Hastie, T. Regularization and Variable Selection via the Elastic Net. *J R Stat Soc Series B Stat Methodol* **2005**, *67* (2), 301–320.
